# The Doctor and the Snake

**Published:** 1985-07

**Authors:** Philip Radford

**Affiliations:** Retired General Practitioner, Bristol


### The Doctor and the Snake

**Philip Radford, M.B., Ch.B., D.C.H.**
Retired General Practitioner, Bristol

The goddess of health, Hygieia, was much venerated, understandably, in the ancient Greek world; her numerous statues usually represented her holding a serpent. Hygieia's father was Aesculapius, the god of medicine, who developed his clinical skills as physician to the Argonauts and made use of herbs in treatment; the serpent and the cock were considered sacred to him. So the Greeks, when wanting a medical cure, sacrificed on altars erected in the shrines of Aesculapius. In those times, the snake was thought of as being wise and statues of Aesculapius showed him holding a staff entwined by a serpent; such a staff, of course, has remained a medical emblem to the present day.

Those who were gods, apparently, commonly had power over serpents, as was the case with Jupiter's son Hercules. Juno sent two snakes to destroy him when he was a child, but these were just seized in his two hands and crushed to death. Clearly snakes were considered to have remarkable powers, yet they continued to be thought to have enigmatic qualities. It is hardly surprising that in the Book of Genesis we read that the serpent was '. . . more subtil than any beast of the field . . .'; indeed, the serpent beguiled Eve and, as punishment, it was decreed for the snake that 'upon thy belly thou shalt go, and dust shalt thou eat . . .'.

While snakes have been revered in the past, many people appear to fear them now, perhaps instinctively. In Britain, the only poisonous snake, the viper *Vipera berus*, is commonly chased and beaten to death, should one be discovered. In the same way, a harmless grass snake *Natrix natrix* is often killed by people who imagine it to be dangerous to them or to children; such people make no attempt to identify the snake or even question whether it has a poison apparatus.

It was observed that snakes sloughed their skins and that the new skin, when revealed, was cleaner and more brightly coloured than the old: the snake moved off as though it had been re-born. The Roman author Pliny maintained that a shed snake-skin was helpful in labour, so during childbirth a sloughed skin was applied to the abdomen. It followed that adder broth and flesh was prescribed as treatment in Britain for many illnesses during the Middle Ages and even up to Georgian times; if a snake shed its old skin and then glided off rapidly into cover then, presumably, the reptile had extraordinary abilities in physical recovery. So it was reasoned that the eating of a snake should impart some of its characteristics to man. Similarly, certain African tribesmen who yearn for extra strength and speed eat the meat of the lion *Felis leo* or the leopard *Felis pardus*.

Snake venom is a potent protein mixture and, as is well known, the venoms of some species do have medical applications. Vipers produce venom which is both haemolytic and cytotoxic: after a bite, pain, local swelling and eccymosis result. The poison is injected subcutaneously through the canals of the adder's fangs; these paired fangs are folded back along the roof of the mouth when not in use. The fangs are erected as the snake strikes and, if they are damaged, replacements are grown. Venom is produced by the snake's parotid glands; it should be remembered that even very young adders can both prepare venom and bite and, further, several repeated bites, still involving significant volumes of venom, are possible from juveniles as well as adults.

In Britain, adders occur in Scotland, Wales and England, being found on heaths and in woodland glades. Adders do not attack man unless disturbed or threatened. Such disturbance is often accidental, when a person nearly steps on a snake or almost touches one when picking blackberries or wild flowers. So, in an adder-infested area, it is sensible to refrain from walking barefoot and to be vigilant when examining low vegetation. Nevertheless, vipers normally just writhe and slide away into cover when humans come towards them, especially whey they are muscularly active after being warmed in the sun.

Should a person be bitten by a viper, it is better to stay still so that the toxin is not spread by muscle contraction. The puncture marks of the fang tips will be visible and, as the venom has already been injected and as the toxins are soon fixed in the tissues, incision is not indicated and neither, in my view, is the use of a tourniquet. It is the hand or foot which is most commonly attacked but, while very unpleasant, a bite from an adder is rarely fatal in Britain; hypersensitivity to the horse serum of the antivenom is often a greater problem than the bite.

For adder bites, it is probably unnecessary to use antiserum but, with severe venom reactions, steroids are valuable. Hydrocortisone 100 mg by injection should be given and a broad-spectrum antibiotic will help prevent secondary infection. Immunisation against tetanus should be carried out, the method depending on the immune state of the patient. Snake-bite is a frightening event for most victims, hence it is advisable to give a sedative in addition to analgesics as necessary.

Doctors know of the intense fear which many people have of the snake and the snake-form, amounting to a phobia in extreme cases. Some individuals, usually females in my experience, seem equally afraid of adders, grass snakes, slow-worms *Anguis fragilis*, common eels *Anguilla anguilla* or conger eels *Conger conger*. Doubtless there are sexually-based psychological explanations of the condition, but I have not been impressed by the results of psychoanalysis. Most patients are prepared to continue to live with their snake phobia for, after all, town dwellers and even many country people do not encounter snakes at all frequently and most sufferers will agree that this is the case.

One defence reaction of snakes, when cornered, is to hiss. Humans will hiss and boo at stage actors who do not come up to expectations and domestic cats will spit and hiss when in aggressive mood. Then certain birds, notably geese, swans and owls hiss at aggressors, especially if their young are in danger. Perhaps more significantly, several small cavity-nesting bird species will hiss at an intruder at the nest-hole; as an example, a blue tit *Parus caeruleus* will emit hissing noises should a wood mouse *Apomedus sylvaticus*, for instance, disturb an incubating bird. Snakes are enemies of mice and it could well be that the evolution of a hiss by a bird has protective value. Certain bird species, as well as mice, will avoid snakes if possible; this can be tested by displaying model snakes.

While the modern doctor is unlikely to prescribe adder broth for his patients, he may seek relaxation by observing vipers or grass snakes in their natural habitats. Vipers, with a dark zig-zag patterning down the back and an inverted V on the head, emerge from their hibernating holes in April; normally the pale males are seen first and the larger, red-brown females come out one or two weeks later. Both sexes bask on slopes in the available sun, but they are usually very wary of any human approach. Adders have no organ of hearing as the tympanum is absent, but no doubt they detect ground vibrations which are transmitted through the jaw bones.

If suspicious and alerted, the adder protrudes and flicks its forked tongue by which it senses the air and can detect the scent of an enemy; thus, if one is looking for adders, it is as well to take care that one's scent cannot be blown towards them. Moreover, vipers have effective vision by using their small eyes; the pupil is slit vertically, like that of a cat, and this suggests an adaptation to good vision in twilight or as in the shade of herbage or hollow dens.

After the males have shed their skins, mating occurs, the males being attracted by pheromones from the females' anal glands; the pregnant females give birth to their young in late August in Britain. Vipers start to feed actively after they have moved away from their hibernating areas, following skin sloughing; common lizards *Lacerta vivipara*, mice, bank voles *Clethrionomys glareolus*, short-tailed voles *Microtus agrestis* and young birds are all eaten. A viper will surprise and then bite a small mammal; in due course the venom takes effect and the animal dies. Detecting scent with its tongue, the viper will follow the poisoned animal at leisure; often the prey is swallowed in a hole or in thick undergrowth. Vipers can propel themselves very quickly into herbage if in danger, but they cannot keep up such a speed for long; this is not surprising as the single functioning lung is relatively small. Hence it is helpful to the species that there is no need to rush when in pursuit of poisoned prey. In late summer, adders return to their hibernating areas, usually facing to the south, and most will disappear below ground during October.

Grass snakes, with distinctive yellow collars, are larger than vipers and spend more time near water. They swim frequently and obtain much of their prey in water; frogs *Rana temporaria*, newts *Triturus vulgaris* and small fish are captured and swallowed. Unlike vipers, grass snakes lay leathery-coated eggs from which their young are hatched. Eggs are laid in moist, warm places where vegetation is rotting: a compost heap is a favourite site.

Vipers and grass snakes are taken by other animals in the wild although to what extent the potential predators are bitten by adders is uncertain. Red foxes *Vulpes vulpes* and badgers *Meles meles* will, at times, eat both species; further, it is not uncommon for dogs or cats to worry vipers and it is then that they get bitten. Presumably the same could happen to adder-teasing foxes. I have seen a buzzard *Buteo buteo* carrying a snake in its talons and, by its large size, this was probably a grass snake; I expect that the bird-of-prey would have swooped down to secure the snake while it was sun-bathing.

Again, I once watched a kestrel *Falco tinnunculus* which was flying with a small, dark snake; it is likely that this was a viper but whether the snake had been killed already or whether it was poised to strike at the bird with its fangs to try to gain its release is an unanswered question. Maybe the snake's fangs could not penetrate through the bird's feathers, but protection must depend on the total feather thickness at the site of the strike.

It seems that the study of snakes, directly or indirectly, has much to interest the doctor. This applies in a limited way even in Britain where the number of snake species is minimal and there is only one poisonous variety. But observation and investigation of snake behaviour and biology is surely important and much remains to be discovered.

Snakes are prominent in folklore and mythology; in addition, the snake has significance in psychological analysis and psychiatry. Furthermore, the complex chemistry and pharmacology of snake venoms is a big and far-ranging subject; the varied compounds are potent and could well yield drugs of future medical value, as well as continuing to make available substances with known therapeutic properties and which require further assessment.

Nonetheless, people who may have general agreement with these views and who, seemingly, are entirely rational in their outlook, often become upset when confronted suddenly with a snake. In the Book of Numbers there is the passage: '. . . the Lord sent fiery serpents among the people, and they bit the people . . .'; many persons today must be happy that they do not live under those snake-infested conditions. Further, I have no doubt that the doctors amongst them are grateful that snake-bite is a condition which is not encountered often in Britain at the present time, especially as patients' eager friends often bring along dead, aggressor snakes for inspection.

